# Inclusion of pregnant women in antiretroviral drug research: what is needed to move forwards?

**DOI:** 10.1002/jia2.25372

**Published:** 2019-09-16

**Authors:** Lee Fairlie, Catriona Waitt, Shahin Lockman, Michelle Moorhouse, Elaine J. Abrams, Polly Clayden, Marta Boffito, Saye Khoo, Helen Rees, Amandine Cournil, Willem Francois Venter, Celicia Serenata, Matthew Chersich

**Affiliations:** ^1^ Wits Reproductive Health and HIV Institute Faculty of Health Sciences University of the Witwatersrand Johannesburg South Africa; ^2^ Department of Molecular and Clinical Pharmacology University of Liverpool Liverpool United Kingdom; ^3^ Infectious Diseases Institute Makerere University College of Health Sciences Kampala Uganda; ^4^ Brigham and Women's Hospital Harvard T.H. Chan School of Public Health Boston MA USA; ^5^ ICAP at Columbia University Mailman School of Public Health and Vagelos College of Physicians and Surgeons Columbia University New York NY USA; ^6^ HIV i‐Base London UK; ^7^ Chelsea and Westminster Hospital London UK; ^8^ Unité Mixte Internationale 233 Institut de Recherche pour le Développement U1175‐INSERM University of Montpellier Montpellier France

**Keywords:** HIV, teratogen, drug dosing, maternal health, child health, antiretroviral, pregnancy, dolutegravir, birth defects

## Abstract

**Introduction:**

To adequately ascertain drug safety and efficacy, drug trials need to include participants from all groups likely to receive the medication following approval. Pregnant women, however, are mostly excluded from trials, and women participating are often required to use highly effective contraception and taken off study product (even off study) if they conceive. There is little commercial incentive for including pregnant women in clinical trials, even when preclinical animal and human pharmacokinetic and safety data appear reassuring. With this conservative approach, large numbers of pregnant women are exposed to drug postlicensing with little known about drug safety and efficacy, and little done to systematically monitor outcomes of pregnancy exposure.

**Discussion:**

The article focuses on antiretrovirals for treating and preventing HIV, and presents potential approaches which could extend to other therapeutic areas, to obtaining adequate and timely data to inform use of these drugs in this population. Most importantly the pregnancy risk profile of investigational agents can be systematically stratified from low to high risk, based on guidelines from regulatory bodies. This stratification can determine the progress through preclinical work with animals and non‐pregnant women to opportunistic studies among women who become pregnant on a clinical trial or within routine clinical treatment. Stratification can include pregnant women in clinical trials, concurrent with Phase II/III trials in non‐pregnant adults, and ultimately to postmarketing surveillance for outcomes in pregnant women and their infants. Each step can be enabled by clear criteria from international and local regulatory bodies on progression through study phases, standardized protocols for collecting relevant data, collaborative data sharing, pregnancy outcomes surveillance systems supported by committed funding for these endeavours.

**Conclusions:**

A formalized step‐wise approach to including pregnant women in antiretroviral drug research should become the new norm. Systematic implementation of this approach would yield more timely and higher quality pregnancy dosing, safety and efficacy data. Through more vigorous action, regulatory bodies could responsibly overcome reluctance to include pregnant women in drug trials. Funders, researchers and programme implementers need to be galvanized to progressively include pregnant women in research – the use of newer, more effective drugs in women is at stake (349).

## 1 Introduction

Globally, antiretroviral (ARV) drug use in pregnancy has evolved from single and two‐drug regimens for prevention of vertical infection, to three‐drug regimens for all pregnant and breastfeeding women, to protect their own health in addition to preventing vertical (and horizontal) transmission 1, 2, 3. Underpinning this change is a drive towards a simplified and harmonized “public health” approach, with a single regimen across all age groups and populations. This simplifies HIV treatment programmes; minimizes prescribing errors; simplifies drug manufacture and supply chains; and ultimately improves treatment access. However, relatively few studies have evaluated drug safety of combination three‐drug antiretroviral (ART) regimens in pregnant and breastfeeding women. Women are under‐represented in ART clinical trials in general, making up only about 20% of participants 4, and are usually discontinued from a trial (or at least from study drug) if they become pregnant. Thus, relatively little is known about the teratogenicity, safety, pharmacokinetics, dosing and efficacy of many ARVs in pregnancy 2, 3, 5. A number of new ARVs have become available in recent years which have improved tolerability, barriers to HIV drug resistance, virologic efficacy (in some instances) 6, 7, and formulations, including long‐acting injectable formulations 8, 9. Gaps in data may result in a substantial delay – frequently years or decades – before these drugs become available to pregnant women, or may even delay use of these drugs in the general population. In some instances drugs are used without adequate pregnancy data. These challenges are not restricted to ARVs. Over half of pregnant women are prescribed at least one medication and almost all take over‐the‐counter medications, mostly under‐studied in pregnant women 10, 11, 12.

Historically, pharmaceutical companies, researchers and funders have taken a conservative approach to including pregnant women in trials. Progress in changing this approach has been slow, despite regulatory bodies such as the United States (US) Food and Drug Administration (FDA), Council for International Organizations of Medical Sciences (CIOMS) in collaboration with the World Health Organization (WHO), and ethicists laying out recommendations for inclusion of pregnant women in research (Box 1) 11, 13, 14. In addition, the National Institutes of Health requires review of all submissions, for inclusion of women and children; although not pregnant women specifically 15. Frequently, once drugs are registered in adults, studies in pregnant women do not follow even when postmarketing safety data from women who have conceived on or taken the product during pregnancy raise no safety concerns. Little financial incentive for drug companies, the existing regulatory and funding environment, together with concerns regarding litigation if any adverse outcomes occur 16, have discouraged researchers and clinicians from conducting studies or surveillance of new drugs in pregnancy. Guidance from the WHO, US FDA, European Medicines Agency and local regulatory agencies about the *type and amount of safety data* required before ARVs can be used in clinical trials involving pregnant women or women of childbearing potential (WOCP), is lacking, and would go a step further in stratifying risk and including pregnant women in different phases of studies. These bodies could also provide considerations on legal and litigation aspects of including pregnant women in research.

Box 1 **International Regulatory and Ethical framework for including pregnant women in research**

1 Draft guidance from the **Food and Drug Administration (FDA) in 2018** highlighted several important reasons for including pregnant women in research, including the need for safe and effective drugs in pregnancy; the need for adequate safety, efficacy and dosing data; and; the possibility of access to benefits for the mother and fetus that are not available outside the research setting 14. The report notes unequivocally that access of pregnant women to treatment options is a significant public health issue. The guidance recommends the inclusion of pregnant women in clinical research in the following circumstances: post‐marketing (drug is FDA registered) where adequate nonclinical data are available, including on pregnant animals; where adequate safety data in non‐pregnant women are available, or “preliminary safety data from the literature or other sources regarding pregnant women” are available; where efficacy cannot be extrapolated to pregnant women, and safety cannot be determined without a clinical trial 14. For pre‐clinical settings or investigational drugs, nonclinical studies must be conducted first and if pregnant women are included there must be the prospect of direct benefit for the mother and/or fetus from participation in the clinical trial, not obtainable outside the clinical trial or by another mechanism. Informed consent must be obtained by the pregnant woman in all cases; however, where direct benefit is solely for the fetus, informed consent from the father is also recommended, apart from circumstances where this is not possible or desirable 14.

The **CIOMS** recommends that women of child‐bearing potential are only included in clinical research if access to pregnancy tests and termination of pregnancy (TOP) is possible. As , this may restrict research in many countries where TOP is not permitted, but where the investigational drug could be of benefit. In these situations, research is recommended if there is a compelling social benefit for research and if alternative means of accessing TOP is possible.

The CIOM guidance recommends that in research including pregnant women, risk should be no more than minimal, and that the research is conducted to provide information directly related to pregnant women and their foetuses. Further, a research committee may consider including women in research with a slightly greater than minimal risk, where there is social value for inclusion and where the research cannot be done on non‐pregnant women 13.

**Bioethicists** propose **3 key principles** in support of including pregnant women in research – the woman's right to **effective, safe and equitable access** to treatment 11. A recent Task Force reporting directly to the US Secretary for Health and Human Services and Congress, highlights that although randomised controlled trials are considered the “gold standard” of drug research, these studies rarely yield findings specific to pregnant women, who can be considered “drug orphans” [17, 18]. The Task Force suggested a number of ways that data can be obtained from pregnancy including: pharmacokinetic or pharmacodynamic studies in pregnant women, expanded access to surveillance mechanisms such as registries, post‐marketing surveillance, post trial data; strengthen evidence base on the impact of common disease on pregnant women, and increasing the number of controlled trials evaluating the risks of using drugs during pregnancy 18

Against this backdrop, we discuss issues related to conception while taking ARVs or ARV initiation in pregnancy, present case studies illustrating key challenges, and propose an approach to addressing data gaps.

## 2 Discussion

### 2.1 Teratogenicity

Teratogenicity is the impact of factors including environmental, microbial, drug, radiation or chemical exposures causing abnormal development in the foetus through genetic (single gene affected) or chromosomal (a number of genes affected) abnormalities, vascular or mechanical changes 19. The greatest risk is between three to eight weeks of development, ending by the 13th week of gestation when organogenesis is complete 19, 20. Teratogenicity is at the forefront of safety concerns around new drugs in pregnancy. Animal models have traditionally been used to detect signals for teratogenicity. However, given the interspecies variability in responses to drug exposure and unknown predictive value of animal studies for humans, translation of animal teratogenic risk to humans is difficult to predict 20, 21. Indeed, the difficulties with interpreting animal data may have untoward effects, as shown with efavirenz, where animal data, together with a few isolated human case reports suggested a risk of neural tube defects (NTDs). These concerns had a major programmatic impact that lasted for seven years when sufficient evidence had been gathered on the drug's safety (Box 2).

Box 2 **Case scenarios**

**Efavirenz and teratogenicity**

NTDs occurred in 3/20 cynomolgus monkey fetuses exposed to efavirenz, compared to 0/20 in unexposed monkeys 22. An increase in congenital anomalies were suspected following four case reports of NTDs in children exposed to efavirenz at conception 23, 24, 25. As a result of the monkey data, in 2005, the FDA changed the grading of efavirenz from C (animal studies have shown harm and human studies have not demonstrated safety) to D, denoting risk to the fetus of drug exposure 23. This led to a recommendation from WHO and other guideline bodies, that all women of childbearing potential or in the first trimester of pregnancy receive nevirapine‐based ART, while all men and women not of childbearing potential received efavirenz 26. Nevirapine was believed to carry high risks of hepatotoxicity and Steven Johnson Syndrome, particularly in women with a higher CD4 count > 250 cells/mm^3^, which were not necessarily balanced against an unclear teratogenicity risk 27, 28. The lack of a universal regimen posed numerous programmatic challenges, such as different supply chains, complexity of nevirapine administration including the 2‐week lead in dose, inability to co‐dose with rifampicin, monitoring of liver function and a twice daily compared to once daily regimen. Subsequently, a series of meta‐analyses found that, compared to women receiving non‐efavirenz‐based regimens, there was no increase in congenital anomalies, particularly NTDs 29, 30, 31. Data also showed that efavirenz was superior to nevirapine in clinical, virological and adherence outcomes 32, 33. Further, a modelling study from Côte d'Ivoire indicated that, compared to nevirapine, the long‐term survival benefit in women receiving efavirenz at 10 years of follow‐up was much higher than the small, if any, risk of teratogenicity in exposed infants 34. Thus, supported by the above data, in 2012, WHO changed its recommendations to include efavirenz for all adults, including women of childbearing age and pregnant women, and in so doing unified the adult – and indeed child – ART regimen 33, 34.

Clearly, the issue of potential teratogenicity presents a dilemma in clinical trials that include women who wish to conceive or are in early pregnancy (<13 weeks). It is not ethically acceptable to conduct randomized ARV trials in women who intend to or are at risk of conceiving, with the primary aim of ascertaining safety of exposure to a drug in the periconception period. Even if it were, gathering sufficient data on individual defects would require recruitment of enormous numbers of women to adequately power the study. However, it is possible to systematically collect relevant data through two means. First, outcomes can be evaluated in women who become pregnant while taking part in ARV trials. The numbers of pregnancies assessed will likely be insufficient for evaluation of rare birth defects and the occurrence of isolated cases of defects may result in responses similar to that described with efavirenz. Second, surveillance programmes can prospectively monitor teratogenicity in infants exposed to ART at conception and in the first trimester of pregnancy. To minimize a range of potential biases, these data need to be collected systematically, prospectively and in a large population of women with a variety of exposures, examples of which follow.

The Tsepamo surveillance study in Botswana is an example of active surveillance, extracting data from maternal records and conducting newborn surface examination to evaluate for congenital anomalies (with review of photographs and descriptions of anomalies by an expert panel that is blinded to exposure group) 49. The study, initially set up to evaluate efavirenz‐associated/related neurotoxicity, has, since August 2014, prospectively collected data on birth outcomes, stratified by HIV‐1 status and ART regimen and including HIV‐negative women as controls, in about 90,000 births in eight centres in Botswana, about 45% of all births in the country.

The French Perinatal Cohort (EPF) prospectively enrols and follows HIV‐positive pregnant women and their infants for two years, and compares outcomes between children exposed to different drugs 50, 51.

The Antiretroviral Pregnancy Register (APR) has a passive surveillance approach, with data collected prospectively by healthcare providers as early as possible in pregnancy, updated with outcomes data, including birth defects and foetal outcomes, but no cohort follow‐up 52. The APR currently includes data from about 15% of HIV‐positive pregnant women in the US and about 350 women from other countries 52, and compares the rate of congenital anomalies to two other US registers: the CDC's birth defects surveillance system and the Texas Birth Defects Registry 53, 54, and outcomes associated with drug exposure in the first trimester compared with second and third trimester exposures 52.

The WHO Pregnancy Registry was piloted in 2010/2011 in Brazil, Ghana, Kenya, Uganda and Tanzania, as a pregnancy surveillance system for low‐ and middle‐income countries (LMICs). It has, however, not been widely implemented and challenges are anticipated with data completeness and validity 54, 55, 56. Together these surveillance studies and registries may identify signals of potential teratogenicity which can be evaluated across all available resources 50, 51.

Protocols have also been developed to evaluate pregnancy and neonatal outcomes in HIV prevention trials. For example, the Microbicide Trial Network (MTN) developed protocol MTN 016 to enrol all infants of women who became pregnant while receiving any of the study products evaluated in MTN studies 57, 58.

As a result of the Tsepamo surveillance, with a National Programme ART regimen change to dolutegravir(DTG) in 2016 in Botswana, investigators unexpectedly found that four infants born to 596 women who conceived on DTG had developed NTDs (0.67% NTD, 95% CI 0.26%, 1.7%), substantially higher than in women conceiving on non‐DTG regimens and in HIV‐negative women 59, 60, 61. These data, reported in 2018, suggest a signal for NTD in women conceiving on DTG. However, interpreting these findings in a single population, for a rare event, is problematic; hence more data, from different settings, are required to evaluate these risks 59, 60.

Overall, surveillance provides opportunities to evaluate teratogenicity in pregnancy, but requires an accurate denominator and a high retention rate, contingent on adequate funding and health systems capacity, and motivated healthcare workers. However, the surveillance data from Tsepamo highlight that there is much uncertainty in how to confirm signals that are identified in other studies, to extrapolate findings when they are only detected in specific populations and to make policy recommendations regarding the use of these drugs based on limited data. This uncertainty may lead to women receiving different, less effective drugs with more side effects than their male partners, undoing hard work done to achieve a universal regimen 62. The most notable previous example of this was the practice of prescribing nevirapine for women and efavirenz for men until 2012 (Box 2). A similar approach is being taken in some countries with DTG until more data become available, meaning that many women who are pregnant, breastfeeding or WOCP will not have access to this drug, despite WHO recommendations that DTG be part of first‐line treatment in pregnant women after eight weeks gestation and in non‐pregnant women who have access to effective contraception 63. Some countries have elected to provide DTG to women regardless of concerns about teratogenicity, others only recommend DTG if good contraceptive services and good reproductive healthcare can be “guaranteed” 63, 64. These recommendations have raised questions about equity, inclusion of all stakeholders in decision‐making processes, and communication of risks and benefits of drug options to women, regarding pregnant women 65. A recent modelling paper by Dugdale *et al*., reports that despite the possible increased risk of NTD compared to efavirenz, DTG is likely to improve maternal mortality outcomes and reduce HIV transmission overall, arguing against DTG avoidance in WOCP 66.

### 2.2 Maternal and infant safety

Maternal safety concerns directly related to pregnancy include haemorrhage, hypertension, sepsis, abortion, peripartum depression, hepatic, haematologic and renal disease. Infant safety concerns encompass prematurity, low birth weight, stillbirth, toxicity secondary to maternal drug or toxin exposures, neonatal jaundice, infections and hypoxic/ischaemic events, and HIV drug resistance in HIV‐positive children. Poor maternal outcomes are still prevalent across many LMICs, with an increased risk related to factors such as HIV disease and complications such as TB and anaemia, common in these settings 67. In observational studies, maternal HIV infection and ART have been associated with preterm delivery, low birth weight, small for gestational age infants and stillbirth, compared with HIV‐negative women 49, 53, 68, 69, 70. In most LMICs, there is a paucity of data describing background pregnancy outcomes apart from broad indicators such as maternal death and stillbirth, making it difficult to directly compare outcomes related to HIV and ART to a population norm 71.

Furthermore, there are considerable challenges with evaluating maternal and infant safety outcomes illustrated in Box 3. The first challenge is determination of causality related to a specific maternal and infant safety outcome. Given that HIV‐positive pregnant and peripartum women, and their infants already have an increased risk for adverse events, causality related to a particular ART regimen may be difficult to prove (particularly in observational studies), confounding analysis and interpretation. An example described below illustrates what happened with maternal nevirapine and severe rash and liver toxicity, with particular concern in women with “higher CD4 counts” >250 cells/mm^3^ (Box 4) 27, 28.

Box 3 **Case scenario**

**Difficulties regarding interpretation of infant safety in the PROMISE study**

The PROMISE/IMPAACT P1077 study enrolled 3490 HIV‐positive pregnant women at a median 26 weeks gestation, all of whom had a CD4 count >350 cells/mm^3^ 3. Women were randomised to receive zidovudine alone with a tenofovir and emtricitabine tail, or triple therapy containing lamivudine, lopinavir/ritonavir, and either zidovudine or tenofovir as the third agent. The dose of lopinavir/ritonavir was doubled in the third trimester, based on data from pharmacokinetic studies evaluating lopinavir/ritonavir in pregnancy in women (opportunistic studies) 35, 36, 37. HIV transmission rates were lower in the triple therapy arms (0.5%) than the zidovudine arm (1.8%) (difference −1.3%, CI −2.1 to −0.4). However, low birth weight was more common with zidovudine‐based (23.0% vs. 12.0%, *p*<0.001) or tenofovir‐based ART (16.9% vs. 8.9%, *p*=0.004), compared to zidovudine alone. Preterm delivery was more common with zidovudine‐based ART compared to zidovudine alone (20.5% vs. 13.1%, *p*<0.001), and very preterm delivery (<34 weeks; 6.0% vs. 2.6%, *p*=0.04) and early infant death more common in tenofovir‐based ART compared to zidovudine‐based ART (4.4% vs. 0.6%, *p*=0.001). These findings have not been fully explained and it has been postulated that these outcomes were related to an unknown confounder in the zidovudine group, or to interactions between tenofovir and lopinavir/ritonavir, particularly given the higher lopinavir/ritonavir dose 3. This case highlights the difficulties in attributing causality of ART regimens to infant safety outcomes.

Box 4 **Case scenario**

**Nevirapine and maternal safety**

In the early 2000s there were several reports of increased rash and liver toxicity (up to 11 times higher) in pregnant women taking nevirapine, particularly those with a CD4 count >250 cells/mm^3^ 27, 28. Other studies suggested that the risk of liver toxicity in pregnant women taking ART in general was higher than in women overall 38, 39, 40, 41, 42, 43, 44. Caution and close monitoring, with liver function monitoring, was advised, particularly for women with CD4 counts >250 cells/mm^3^. This increased the programmatic complexity of guidelines at a time where few alternatives were available, particularly in pregnancy. Subsequently, however, a number of studies, and a systematic review of nevirapine‐associated toxicity in pregnancy, have reported no higher association in pregnancy compared to the general population, and liver toxicity appears to be of lesser concern than previously thought 45, 46, 47, 48. In summary, until 2012, despite the considerable safety concerns regarding nevirapine and the additional programmatic complexity, its use was favoured over efavirenz in pregnant women, especially in the first trimester. Both the toxicity and teratogen concerns were later refuted.

The second challenge is making diagnoses of adverse events, particularly where accurate gestational age is required, such as prematurity and small for gestational age infants. Ideally gestational age should be based on early foetal ultrasound correlated with last known menstrual period using guidelines such as those of the American College of Obstetricians and Gynaecologists 72. However, ultrasound is not routinely readily available outside of high‐income countries and research settings, and in LMICs women frequently present to their first antenatal clinic visit after 20 weeks with unreliable menstrual history. Outcomes related to gestation may thus be attributed to ART without accurate gestational ageing or a control group.

The complexities in evaluating safety and interpretation of relatedness to study drug, seriousness and grading of safety events are not unique to HIV and ART outcomes. In maternal vaccine studies, in response to safety concerns, the Global Alignment of Immunisation safety Assessment in pregnancy (GAIA) consortium have developed guidance in which outcomes potentially related to maternal vaccination are graded according to the quality of available data. This assists with forming an appropriate response to any safety signals which emerge and these principles could be applied to ARV research in pregnant women 73.

### 2.3 Antiretroviral pharmacokinetics during pregnancy: implications for efficacy and safety

Physiologic changes during pregnancy alter the absorption, distribution, metabolism and elimination of drugs. These changes have implications for the efficacy and safety of ART during pregnancy, and the risk of HIV transmission to infants, particularly in HIV‐positive women who present to antenatal care late in their pregnancy or are diagnosed with acute HIV infection in pregnancy.

Pregnancy is considered a particularly high‐risk period for HIV‐acquisition in women 74. Pharmacokinetic (PK) changes in pregnancy may lower intracellular tenofovir disphosphate concentrations by between 45% to 58% in later pregnancy, compared to pre‐pregnancy and non‐pregnant women, which could substantially impact on the level of protection afforded by oral tenofovir‐based pre‐exposure prophylaxis (PrEP) 74, 75. More data are required to assess safety and dosing of oral PrEP in pregnancy and whether PK changes impact on prevention efficacy 76. Furthermore, women who become infected in prevention studies should be included in acute HIV studies.

Two approaches are generally used to gather data to address questions of PK in pregnancy, namely “opportunistic studies” in women who become pregnant while taking ARVs and studies specifically designed to evaluate PK in pregnancy. While these studies focus on PK evaluations, they can also provide useful information on maternal and infant drug safety.

In “opportunistic studies,” PK and safety evaluations are done at several time points during pregnancy or postpartum in women receiving ARVs postlicencing, either as part of a clinical trial, or in routine care, usually many years after approval. A number of studies use this approach. The two most notable examples are the IMPAACT P1026s study and the “Pharmacokinetics of newly developed ANtiretroviral agents in HIV‐positive pregNAnt women” study (PANNA). To date, this approach has been successfully used to evaluate over 15 ARVs, in various combinations, including newer ARV drugs as they become available 35, 36, 77, 78, 79, 80, 81, 82, 83, 84, 85, 86, 87, 88, 89, 90, 91, 92, 93, 94, 95. The main drawback of this approach is the delayed availability of data, frequently years postlicencing/registration for newer and better drugs in pregnancy.

Some studies have allowed women who become pregnant to continue on the study drug, and conducted additional PK evaluations on these women. However, the number of evaluable women tends to be fairly small 96. Overall, opportunistic evaluations can provide the basis for dosage recommendations in pregnancy, raise concerns requiring further prospective study and provide some description of drug safety.

In both treatment and prevention studies, drug exposures in the infant may also be “opportunistically” measured, by doing “washout PKs,” where infants are exposed to maternal ARVs. This provides data which can be modelled before formal PK studies, depending on the suitability of the new ARV to be used for treatment or prophylaxis in this age group 97.

An additional approach to gathering pregnancy PK data would be to conduct small PK/safety studies in pregnant women, either concurrent with Phase III trials in non‐pregnant adults or, in certain cases, concurrent with Phase II trials in non‐pregnant adults. These small pregnancy studies could be stand‐alone, or pregnant women could be included in the Phase II/Phase III trials being conducted in adults. This approach would yield more timely pregnancy PK and safety/efficacy data (from a limited number of women), which could expedite the study and use of newer drugs during pregnancy. The approach may be particularly relevant for newer medications in WOCP, and for which preclinical reproductive toxicity data are reassuring and no other potential pregnancy concerns exist.

**Figure 1:**
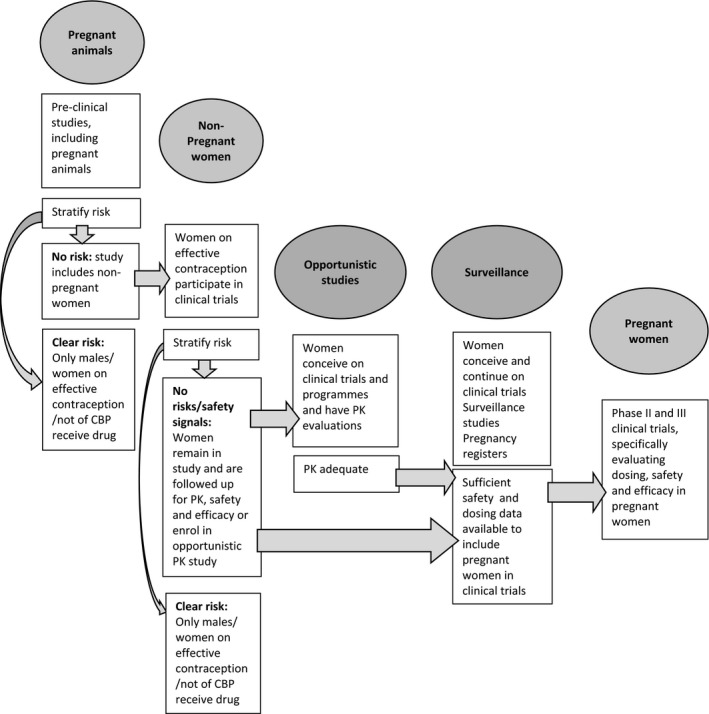
Clinical trial phases and a stepwise approach towards including pregnant women in research. CBP, child‐bearing potential.

In addressing questions of teratogenicity, safety, pharmacokinetics, dosing and efficacy a number of approaches, or enabling factors are required to successfully advance this field and ensure progress. These include clearly formulated recommendations from regulatory bodies and ethicists, and tested strategies (Box 1). Figure 1 represents the different phases of clinical trials, the types of studies that might be included in each phase and Box 5, lists enablers which aim to increase the involvement of pregnant women in clinical trials, or improve the collection of data from women who become pregnant in these studies.

Box 5 **Case scenario**

**Overall enablers to advance research in HIV‐positive pregnant women**

WHO and other International and local regulatory bodyguided pre‐defined thresholds of the number of pregnancy and safety outcomes that are required before:

1. recruitment of women of childbearing potential in phase I, II and III trials (different thresholds for each of phase)
2. allowing continuation of women in trials and programmes who become pregnant while receiving the ARV of interest
3. recruitment of women wishing to become pregnant in phase I,II and III trials.

These recommendations should explicitly balance the concerns around safety of trial participants, litigation and the real public health harms of an overly‐conservative approach.

Womens' agency in the process needs to be increased. WHO and representatives of civil society and other organisations that promote women's rights, need to be involved in decision‐making regarding the inclusion of women in research. In collaboration, a standardised informed consent form could be developed to be used in the different scenarios (Figure 1), and which give women the choice to, for example, continue in a trial if they become pregnant.

Legislation for funders, mandating that all phase II/III ARV studies need to consider inclusion of pregnant women, and at least have a plan to do so once preliminary safety data is available.

Collaborative arrangements between trial sponsors, pharmaceutical companies, researchers and programme managers to allow data to be rapidly collected and pooled for analysis.

Consideration should be given to making it mandatory that data on all women who become pregnant in phase I, II and III trials are reported to a central body. Local ethics and regulatory requirements would be considered and conditions could be stipulated in ethics review procceses and regulatory approvals.

Increased commitment from funders, researchers and clinicians to contribute to existing surveillance studies, systems and pregnancy registers or establish these where not currently available.

Pooling of data through open access data sharing between different registries and studies, allowing for timely assessments of whether pregnant and breastfeeding women can be included in studies. Data in registries, trials and in routine care should be collected with standardised tools on an agreed set of variables, each with pre‐defined categories. This would include maternal ART exposure, pre‐existing conditions, pregnancy‐related conditions, and exposure to concomitant medications, alcohol, recreational drugs and tobacco. A fetal ultrasound would be preferable for gestational ageing, where possible in the first trimester for gestational ageing, and then around 18‐24 weeks for accurate congenital anomaly screening 98.

Attention to data management issues to ensure clean, complete data sets is important for combined data to be successfully used.

Given the marked variation in quality of data collected, a tiered approach to evaluation of data should be developed, similar to the GAIA consortium recommendations.

## 3 Conclusions

In conclusion, in order to address questions of teratogenicity, safety, PK, dosing and efficacy, a formalized, standardized, step‐wise approach is required for the inclusion of pregnant women in ARV research. At the very least, PK for dose optimization should be done in pregnant women early in drug development, concurrent with phase II /III trials and not only in phase IV studies, especially if the drug is likely to be used in pregnancy postlicencing and if there are no safety signals which should exclude pregnant women. Studies of new ARV drugs need to predominantly include women in LMICs. An evidence and expert opinion‐based guidance, set out by WHO and international and local regulatory bodies would go a long way to clarifying this complex field, and securing the safety and efficacy of ART for women and their infants. These guidelines need to make specific recommendations on the absolute numbers and conditions under which studies can systematically progress through preclinical work with animals and non‐pregnant women, to surveillance mechanisms and opportunistic studies among women who become pregnant on a clinical trial or within programmes, to finally including pregnant women in clinical trials and studying the drug of interest within programmes. Researchers and funders need to be galvanized to act: the use or non‐use of newer, more effective drugs in women is at stake (2996).

## Competing interests

No competing interests to declare.
